# Label-free quantitative proteomics analysis of sea cucumber (*Apostichopus japonicus*) from different origins using data-independent acquisition mass spectrometry

**DOI:** 10.1016/j.fochx.2026.103666

**Published:** 2026-02-13

**Authors:** Qiong Wu, Xiliang Yu, Shiwei Wan, Houde Cai, Jian Jiao, Jiaojiao Shi, Xiuping Dong

**Affiliations:** a State Key Lab of Marine Food Processing & Safety Control, National Engineering Research Center of Seafood, School of Food Science and Technology, Dalian Polytechnic University, 116034 Dalian, PR China; b Beijing Tong Ren Tang Health (Dalian) Seafoods Co. Ltd., 116045 Dalian, PR China; c Shanghai Applied Protein Technology Co. Ltd., 201100 Shanghai, PR China

**Keywords:** Sea cucumber, Data-independent acquisition, Geographical traceability, Food authentication, Proteomics

## Abstract

Sea cucumbers (*Apostichopus japonicus*) from Liaoning, China command premium market value due to superior collagen integrity, yet the proteomic basis of this geographical differentiation remains unclear. Current geographic proteomic studies using data-dependent acquisition (DDA) mass spectrometry face stochastic ion selection bias, poor low-abundance reproducibility and limited coverage. Here, we established a custom label-free data-independent acquisition (DIA) workflow to characterize proteomic profiles of 200 sea cucumbers (80 Liaoning, 120 non-Liaoning). Our approach identified 6278 proteins and discovered 64 differentially expressed proteins (DEPs), including arginase (A0A141R8A8) and glutamine synthetase (A0A076VCY3) as potential authentication markers. Functional annotation highlighted arginine biosynthesis (*P* = 0.00034) as the pivotal pathway, alongside two-component system and necroptosis, indicating nitrogen homeostasis prioritization and stress adaptation in Liaoning specimens. This study provides a novel DIA-based framework for discovering the proteomic basis of geographical differentiation, linking nitrogen metabolism to regional quality differences in marine foods and identifying potential molecular targets for future authentication efforts.

## Highlights

• A label-free DDA-library based DIA workflow was customized for the analysis of sea cucumber proteome.
• The proteomic differences of 200 sea cucumbers from different origins were analyzed and 6278 proteins were identified.
• Arginine biosynthesis pathway (*P* = 0.00034) drives Liaoning sea cucumber adaptation.
• 64 DEPs linked to nitrogen homeostasis, stress response, and environmental sensing.
• Establishes a proteomic framework for marine food origin authentication.

## 1 Introduction

The sea cucumber (*Apostichopus japonicus*), a commercially vital marine species, possesses a collagen-rich body wall as its primary edible component. It is highly valued in global markets for their nutritional richness and therapeutic properties, particularly in traditional medicine and cuisine in East Asian countries (Hossain et al., 2023). In China, sea cucumbers are mainly distributed in Liaoning, Shandong, and Fujian provinces. Among these, specimens from Liaoning command premium market prices due to their superior texture (e.g., higher collagen integrity) and nutritional profiles, as evidenced by physicochemical analyses (Feng et al., 2021; Zhao et al., 2018). The distinct environmental stressors in the Liaoning waters, where the annual fluctuation of seawater temperature is the largest in China due to the influence of the semi-enclosed basin of the Bohai Sea, may have led to the unique proteomic adaptability of sea cucumbers in this area (Hu et al., 2022). However, the proteomic basis underlying such geographical differentiation remains inadequately explored, hindering robust origin authentication and quality assessment frameworks.

Current studies investigating proteomic variations in marine organisms predominantly rely on the labeling data-dependent acquisition (DDA) mass spectrometry (Feng et al., 2020; Madeira et al., 2020; Sun et al., 2017). While DDA enables targeted peptide identification, its stochastic precursor ion selection introduces inherent biases, leading to poor reproducibility for low-abundance proteins and limited proteome coverage (Domon & Aebersold, 2006; Gillet et al., 2012). These limitations are particularly pronounced in complex matrices like marine biota, where dynamic protein expression ranges and environmental adaptations necessitate comprehensive analytical approaches. In addition, when the sample size is large, the labeling strategy will make the sample processing process very complicated and expensive (Aydoğan, 2024). In contrast, data-independent acquisition (DIA) mass spectrometry systematically fragments all precursor ions within sequential isolation windows, ensuring unbiased quantification and enhanced reproducibility across large sample cohorts (Gillet et al., 2012), however, DIA data analysis is challenging due to the multiplexing nature of DIA ms2 spectra. The most widely used approach to DIA data analysis is to extract ion chromatograms from DIA data using a spectral library constructed from DDA experiments (Bruderer et al., 2017; Guan et al., 2020; Pino et al., 2017). This DDA library-based DIA workflow can further improve proteome depth by utilizing spectral libraries customized for specific organisms, thus partially addressing the incompleteness of generic databases. Despite these advantages, the application of label-free DDA library-based DIA in marine organism proteomics, especially for geographically distinct populations, remains underexplored.

In this study, a label-free DDA library-based DIA workflow was established to conduct a comprehensive quantitative proteomic analysis of a large sample of sea cucumbers (*Apostichopus japonicus*). A total of 200 sea cucumber samples were divided into a “Liaoning” group (*n* = 80) and a comparative “non-Liaoning” group comprising samples from Shandong (*n* = 60) and Fujian provinces (*n* = 60), aiming to resolve molecular markers for the authentication of Liaoning origin against these major alternative sources and elucidate functional pathways driving geographical adaptation. The application of DIA in this method mitigates the stochasticity and limited dynamic range of DDA, and the label-free design facilitates the high-throughput analysis necessary for large-scale biomarker discovery, thus addressing the key limitations of conventional proteomic strategies for food traceability. Therefore, this work would provide a promising framework and a solid data foundation for future research into precise and scalable origin verification of marine products.

## 2 Materials and methods

### 2.1 Samples and chemicals

A total of 200 sea cucumber (*Apostichopus japonicus*) samples were collected from commercial aquaculture farms along the coast of China. The cohort was designed to compare Liaoning specimens against those from the other two major production provinces, Shandong and Fujian. The samples comprised 80 individuals from Liaoning Province (4 sampling sites), and 120 individuals from these defined non-Liaoning regions: 60 samples from Fujian Province (3 sampling sites) and 60 samples from Shandong Province (3 sampling sites). The study aimed to identify proteomic markers associated with this specific geographic origin contrast under representative commercial conditions. Samples were therefore collected from multiple independent farms within each region, which typically employ standardized practices and similar types of formulated feeds. All sea cucumber samples were harvested at the commercial adult stage. The mean body weights were 113.7 ± 26.7 g for the Liaoning group and 82.2 ± 25.0 g for the non-Liaoning group (**Table S1**). An independent two-sample *t*-test confirmed no statistically significant difference in body weight between the groups (*P =* 0.094), supporting their comparability at this commercial developmental stage within the natural size variation of the species. This ensured that the individuals used for comparison were at similar stages of physiological development. Detailed sampling information, including the number of samples, average weight, sampling date and detailed coordinates at each sampling site, was listed in **Table S1**.

All samples were transported on dry ice and stored at −80 °C, and samples underwent only one freeze-thaw cycle prior to protein extraction. Protein extraction was performed within 1 month of collection to minimize potential degradation effects from long-term storage. All chemicals used in this study are recorded in **Table S2**.

### 2.2 Protein extraction and peptide digestion

**Protein extraction.** Approximately 2 g of body wall tissue was excised from individual sea cucumber specimens and homogenized using an MP FastPrep-24 homogenizer. The homogenate was lysed with 500 μL of UA buffer (8 mol/L urea, 0.15 mol/L Tris-HCl, pH 8.0), followed by sonication and boiling for 15 min. After centrifugation at 14,000 ×*g* for 40 min (4 °C), the supernatant was subjected to protein quantification using a BCA Protein Assay Kit (Bio-Rad, USA). Next, 20 μg of protein per sample was diluted in 6× SDS-Loading buffer, denatured at 95 °C for 5 min, and resolved on a 4–20% gradient SDS-PAGE gel (180 V constant voltage, 45 min runtime). Protein bands were visualized by Coomassie Brilliant Blue R-250 staining. In this experiment, equal protein aliquots from all samples were pooled to construct the data-dependent acquisition (DDA) spectral library and quality control.

**Filter-aided sample preparation (FASP).** Peptide digestion was performed following the FASP protocol (Wiśniewski et al., 2009). Briefly, samples were reduced with 10 mM dithiothreitol (DTT) at 37 °C for 1.5 h (600 rpm orbital shaking), followed by alkylation with 20 mM iodoacetamide (IAA) in darkness for 30 min at ambient temperature. The reaction mixtures were transferred to 10 kDa molecular weight cut-off centrifugal filters (Microcon®) and sequentially washed with 100 μL UA buffer (8 M urea, 150 mM Tris-HCl pH 8.0; 3×) and 25 mM ammonium bicarbonate (2×). Trypsin digestion was conducted at 1:50 (*w*/w) enzyme-to-protein ratio for 15–18 h (overnight) at 37 °C, with liberated peptides collected by centrifugation at 14,000 ×*g*.

**Peptide fractionation and desalting.** Pooled digests were fractionated into 10 fractions using a Thermo Scientific™ Pierce™ High pH Reversed-Phase Peptide Fractionation Kit. Individual and fractionated peptides were desalted using C18 solid-phase extraction cartridges (Empore™ SPE Cartridges C18, 7 mm bed diameter, 3 mL bed volume; Sigma-Aldrich). Desalted peptides were lyophilised, reconstituted in 40 μL of 0.1% (*v*/v) formic acid, and quantified via UV absorbance at 280 nm using a NANODROP 2000C spectrophotometer. Prior to mass spectrometry analysis, iRT calibration peptides (Biognosys iRT Kit) were spiked into each sample at a 2:1 (sample: iRT) ratio for retention time alignment.

### 2.3 Mass spectrometry analysis for data-dependent acquisition (DDA) and data-independent acquisition (DIA)

All chromatographic fractions designated for DDA library generation were analyzed using a Thermo Scientific Q-Exactive HF-X mass spectrometer coupled to an Easy-nLC 1200 nanoflow chromatography system (Thermo Scientific). Peptide separation was achieved through a reversed-phase C18 analytical column (Thermo Scientific, ES802, 1.9 μm particle size, 75 μm i.d. × 20 cm length) employing a linear gradient of mobile phase B (84% acetonitrile containing 0.1% formic acid) delivered at a constant flow rate of 300 nL/min.

Mass spectrometric detection was performed in positive ion mode with the following acquisition parameters: full MS scans were acquired over a mass range of 350–1800 *m/z* at a resolution of 60,000 (at *m/z* 200) using an automatic gain control (AGC) target of 1 × 10^6^ and a maximum injection time (max IT) of 50 ms. Dynamic exclusion was set to 10.0 s to minimize redundant fragmentation. Subsequent to each full MS scan, 20 data-dependent MS^2^ (ddMS^2^) scans were triggered based on a predefined inclusion list. For MS^2^ acquisition, precursor ions were isolated with a 1.5 *m/z* window and fragmented using higher-energy collisional dissociation (HCD) at 30 eV normalized collision energy. MS^2^ spectra were recorded at a resolution of 30,000 (at *m/z* 200) with an AGC target of 1 × 10^5^ and maximum IT of 50 ms.

For DIA analysis, peptide samples were processed using the same Q-Exactive HF-X/Easy-nLC 1200 platform configuration. Each DIA cycle comprised one full MS scan followed by 44 sequential isolation windows spanning 350–1800 *m/z*. Full MS scans were acquired in profile mode at 120,000 resolution (at *m/z* 200) with an AGC target of 3 × 10^6^ and max IT of 30 ms. DIA windows were fragmented using HCD at 30 eV with the following parameters: MS^2^ resolution of 30,000 (at *m/z* 200), AGC target of 3 × 10^6^, and automatic adjustment of maximum injection time.

### 2.4 Mass spectrometry data analysis

#### 2.4.1 DDA spectral library construction

The spectral library was constructed exclusively from the pooled peptides of all 200 samples. The resulting DDA data were searched against a UniProt-derived FASTA database (downloaded from http://www.uniprot.org) using Spectronaut™ (v14.4.200727.47784). Search parameters included: trypsin specificity with one missed cleavage allowed; fixed modification of carbamidomethylation (C); dynamic modifications of oxidation (M) and N-terminal acetylation. False discovery rate (FDR) was controlled at ≤1% at both peptide and protein levels using a target-decoy strategy. Only peptides with ≥99% confidence and proteins supported by ≥2 unique peptides were retained, resulting in a library containing 60,593 unique peptides mapping to 8198 protein groups.

#### 2.4.2 DIA data processing and spectral matching

DIA datasets were processed using the same Spectronaut™ platform (v14.4.200727.47784) by spectral matching against the pre-established DDA library combined with the UniProt database. Critical software parameters included: dynamic iRT-based retention time prediction, MS^2^-level interference correction, and cross-run intensity normalization. Post-analysis filtering was applied using a Q-value threshold of 0.01, corresponding to an FDR-controlled cutoff of <1% at both peptide and protein levels.

### 2.5 Bioinformatic analysis

**Data preprocessing, normalization, and missing value handling.**

To maintain data integrity and avoid introducing artificial bias, no missing value imputation was performed in this study. Instead, a stringent filtering criterion was applied: only proteins quantified in at least 50% of samples within each group (Liaoning or non-Liaoning) were retained for subsequent differential expression analysis. This approach ensured that the protein expression values used for statistical comparisons were based on actual measurements across the majority of biological replicates. Intensity normalization was conducted in two steps: first, median normalization within each LC-MS/MS run to correct for variations in total ion current; second, cross-run intensity normalization using global scaling in Spectronaut™ to minimize batch effects. The combination of high proteome coverage (6278 proteins) and this conservative filtering strategy resulted in a dataset with high confidence and minimal missing values.

**Identification of differentially expressed proteins (DEPs).**

DEPs were identified using a two-step approach. An initial screening was performed based on a *t*-test *P* value <0.05 and an absolute fold-change threshold >1.5 or < 0.67. Subsequently, to control the false discovery rate (FDR) across multiple comparisons, Benjamini-Hochberg (BH) adjustment was applied to all *p*-values to generate q-values. Proteins with q < 0.05 were defined as high-confidence DEPs. The primary biological interpretation in this study is based on these high-confidence DEPs. The corresponding BH-adjusted q-values for all proteins are provided in **Table S3**.

**Gene Ontology (GO) annotation.**

To establish functional annotations, protein sequences underwent local BLAST searches using NCBI BLAST+ (v2.2.28) alongside InterProScan homology detection. Resultant matches were subsequently mapped to GO terms through Blast2GO implementation. Final annotations were visualized using bespoke R scripting (v3.6.1).

**Kyoto Encyclopedia of Genes and Genomes (KEGG) pathway mapping.**

Following functional annotation, proteins were cross-referenced against the KEGG database (https://www.genome.jp/kegg/) through online BLAST queries. KEGG orthology identifiers were systematically retrieved and mapped to canonical pathways using KEGG Mapper tools.

**Enrichment analysis.**

Functional enrichment was determined through Fisher's exact test implementation, with the entire quantified proteome serving as the background dataset. Statistical rigour was maintained through Benjamini-Hochberg false discovery rate correction (*α* = 0.05). Only terms demonstrating adjusted *P* values <0.05 were deemed statistically significant.

**Principal component analysis (PCA).**

Post-normalization to total spectral peak intensity, processed datasets underwent multivariate analysis in SIMCA-P (v14.1, Umetrics). Pareto scaling was applied prior to PCA execution to mitigate heteroscedasticity effects while preserving covariance structures.

### 2.6 Quality control (QC)

**Sample run order and batch consideration.**

To mitigate potential batch effects, all 200 individual samples and the pooled QC samples were injected in a completely randomized order. This randomized design across multiple instrument days, coupled with systematic QC monitoring, minimized the impact of run-order or day-to-day variability.

**QC sample evaluation.**

to monitor system stability and experimental reproducibility, QC samples (pooled samples in this study) were interleaved at every 7 test samples throughout the analytical sequence. Consistency across 24 QC replicates was assessed using two orthogonal approaches: coefficient of variation (CV) analysis and principal component analysis (PCA). The median CV of QC samples was ∼30%, while PCA revealed tight clustering of QC replicates (**Fig. S1, Table S4**), collectively indicating minimal technical variability and robust system performance.

**DIA Workflow Validation.**

Three critical parameters were evaluated to validate DIA performance: (i) Data points of chromatographic peak: An average of 6 data points per chromatographic peak (minimum threshold >5 points) was achieved, ensuring sufficient sampling for accurate peak integration (**Fig. S2**). (ii) Peak capacity: All samples exhibited peak capacities exceeding 550, surpassing the typical performance benchmark for nanoLC systems (∼ 200), which confirms adequate separation complexity for deep proteome coverage (**Fig. S3**). (iii) Retention time of iRT: 11 of 11 internal reference peptides in iRT kit were detected and demonstrated stable elution profiles across runs (median RT variation <30%), enabling robust retention time alignment between samples. This consistency allows for direct comparative analysis of LC-MS/MS data from individual samples.

## 3 Results and discussion

### 3.1 Protein identification by label-free DDA library-based DIA

Our custom DDA spectral library comprised 60,593 unique peptides and 8198 proteins. Subsequently, based on this library, a total of 6278 proteins were identified from the current 200 sea cucumber samples using the current label-free DIA proteomics analysis method. In contrast, approximately one order of magnitude fewer proteins (548) proteins were identified in sea cucumbers (*A. japonicus*) purchased from Chinese market using label-free quantitative proteomics by sequential window acquisition of all the theoretical fragment ion (SWATH) acquisition mode (Jiang et al., 2021), and 376 proteins were identified in sea cucumbers (*Stichopus japonicus*) captured in the Yellow Sea of China via the label-free quantitative mode of MaxQuant software (Gu et al., 2022) (**Table S5**). The superior performance of our DDA library-based DIA quantitative approach in identifying sea cucumber proteins, compared to SWATH and MaxQuant-based label-free methods, can be attributed to the inherent advantages of DIA's acquisition principle. Unlike traditional DDA that stochastically selects high-abundance precursors for fragmentation, DIA systematically fragments all precursor ions within sequential isolation windows across the entire mass range (Gillet et al., 2012). This continuous MS/MS acquisition mode minimizes missing values and improves reproducibility by eliminating ion selection bias, particularly crucial for detecting low-abundance proteins in complex matrices like marine biota. While SWATH (a specific DIA implementation) shares similar fragmentation logic, its effectiveness depends heavily on optimized window settings and spectral library completeness. Our DDA-built library specifically tailored to sea cucumber proteomes likely provided more comprehensive reference spectra than generic databases used in SWATH analyses, thereby enhancing peptide identification confidence (Bruderer et al., 2015). MaxQuant's reliance on DDA-derived feature detection may have limited its ability to resolve co-eluting peptides in complex samples, whereas DIA's parallel fragmentation across windows preserves quantitative information for all detectable precursors regardless of abundance.

Notably, the comparable number of protein identifications (6278) between our label-free DDA-DIA workflow and certain labeling-based approaches, for example, Feng et al. (2020) identified 5051 proteins from sea cucumbers harvested from five Chinese origins using tandem mass tag (TMT) labeling proteomic approach, and Sun et al. (2017) identified 4073 proteins from sea cucumber collected from the coast of Shandong Province, China using isobaric tag for relative and absolute quantitation (iTRAQ) labeling proteomics (**Table S5**), highlighting the maturation of current DDA library-based DIA in achieving detection depth comparable to traditional multiplexed labeling strategies. Furthermore, the present label-free strategy has the added benefit of reduced sample processing complexity and cost-effectiveness for longitudinal experiments (Aydoğan, 2024), which is more suitable for situations with large sample cohorts such as this study.

### 3.2 Analysis of differentially expressed proteins (DEPs)

**Fig. 1:**
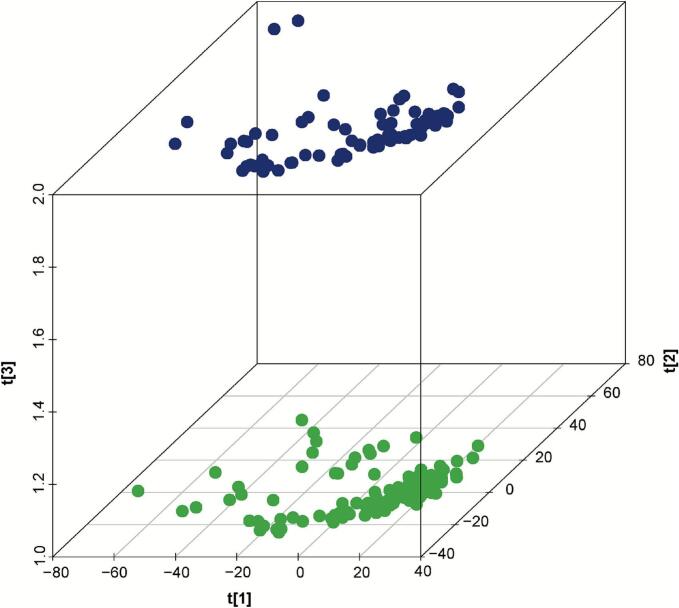
Three-dimensional principal component analysis (PCA) score plot of proteomic profiles from Liaoning (blue) and non-Liaoning (green) sea cucumbers(*Apostichopus japonicus*). (For interpretation of the references to colour in this figure legend, the reader is referred to the web version of this article.)

Following protein identification, we further investigated the differentially expressed proteins (DEPs) between sea cucumber samples of Liaoning and non-Liaoning. Principal component analysis (PCA) in three-dimensional space revealed clear separation in protein expression profiles between the two groups (Fig. 1), with the 2 principal components (PC 1 and PC 2) accounting for substantial variance (47.84% and 20.77%, respectively). This spatial segregation is consistent with the findings of Feng et al. (2020)., highlighting the effectiveness of PCA in discerning geographic variation in proteomes of marine organisms.

**Fig. 2:**
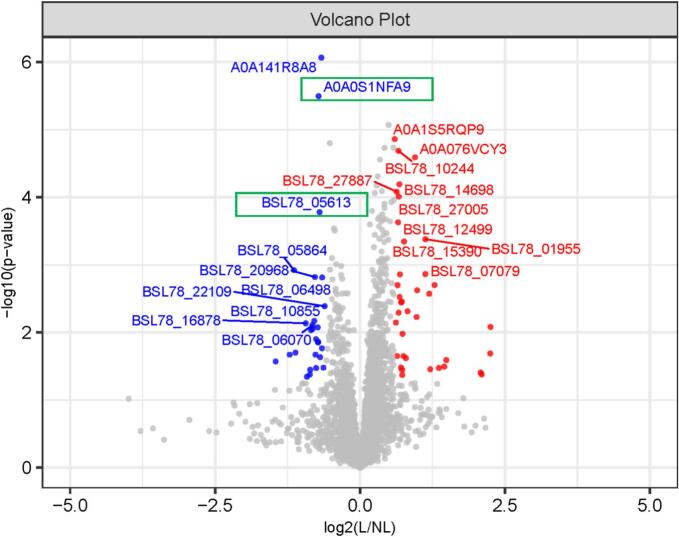
Volcano plot of differentially expressed proteins (DEPs) in Liaoning versus non-Liaoning sea cucumbers (*Apostichopus japonicus*). **Note:** Red and blue dots denote significantly upregulated (fold change >1.5, *P* < 0.05) and downregulated (fold change <0.67, *P* < 0.05) proteins, respectively. Gray dots indicate non-significant proteins. DEPs meet the initial screening criteria, the Benjamini-Hochberg adjusted *P* values for these proteins are provided separately in the **Table S3**. (For interpretation of the references to colour in this figure legend, the reader is referred to the web version of this article.)

Building upon the PCA results that revealed distinct proteomic clustering between Liaoning and non- Liaoning sea cucumber samples (Fig. 1), we identified 64 candidate proteins meeting the initial screening criteria (fold change >1.5 or < 0.67, *P* < 0.05). Following Benjamini-Hochberg FDR correction, 12 of these proteins remained statistically significant (q < 0.05), constituting a set of high-confidence DEPs (9 upregulated and 3 downregulated). These high-confidence DEPs are indicated in bold in Table 1, with the complete dataset provided in **Table S3**. The following discussion focuses on key biological insights derived primarily from this high-confidence set. To align with the exploratory goal of mapping potential biological signals for future hypothesis testing, we generated a volcano plot (Fig. 2) to visualize all 64 initial DEPs. The top 20 most statistically significant DEPs (10 upregulated and 10 downregulated) were specifically annotated to highlight key molecular discriminators (Fig. 2). The volcano plot signatures hinted at some of the potential functional implications of these DEPs. For instance, the ten upregulated proteins in Liaoning samples predominantly belong to the stress-responsive families, including the high-confidence DEPs glutamine synthetase (A0A076VCY3 and BSL78_12499) and cathepsin B (A0A1S5RQP9) (Chen et al., 2017; Kullgren et al., 2013; Shao et al., 2015; Wang et al., 2014). This aligns with previous findings that marine organisms in waters with large temperature differences throughout the year, such as Liaoning waters, frequently upregulate stress-resistance proteins such as heat shock proteins to cope with seasonal temperature fluctuations (Clark & Peck, 2009; Gross, 2004). Conversely, the ten downregulated proteins were enriched in structural components, for example the high-confidence DEP collagenase (matrix metalloproteinase-1) A0A0S1NFA9, fibrinogen C-terminal domain-containing protein BSL78_20968 and cathepsin L-associated protein BSL78_16878 (Chen et al., 2022; Feng et al., 2020; Liu et al., 2016; Zhang et al., 2023). This suppression of structural proteins (e.g., collagenase A0A0S1NFA9) may directly contribute to the high collagen stability and firm texture reported in Liaoning sea cucumbers (Zhang et al., 2023).

**Table 1:** Significantly differentially expressed proteins (DEPs) detected in Liaoning versus non-Liaoning sea cucumbers (*Apostichopus japonicus*) (*P* < 0.05).

Protein ID	Protein Description	Gene Name	Fold change (L/NL)	*P* value
**Upregulated**				
H9TUY5	Ribosomal protein L32e	RPL32e	4.7618403	0.008257152
A0A2G8LA92	Putative neurobeachin	BSL78_05931	4.7456629	0.02040353
A0A2G8L691	Putative long-chain-fatty-acid–CoA ligase 4-like (Fragment)	BSL78_07341	4.2852097	0.041702279
A0A2G8JYL3	Putative deoxynucleoside triphosphate triphosphohydrolase SAMHD1-like	BSL78_22296	4.2336029	0.039073802
A0A2G8L0Q1	DUF1907 domain-containing protein (Fragment)	BSL78_09315	2.8068098	0.025548046
A0A2G8LDD8	Putative transient receptor potential cation channel subfamily M member 2 isoform X6	BSL78_04824	2.7405118	0.031883187
A0A2G8L3G5	Topoisomerase II-associated protein PAT1 (Fragment)	BSL78_08319	2.5692538	0.033456884
A0A2G8JBG1	Putative hydroxyacid oxidase 1	BSL78_30106	2.4389807	0.001983939
A0A2G8JDJ0	Putative delta-1-pyrroline-5-carboxylate synthase isoform X2	BSL78_29372	2.3154717	0.034955639
A0A2G8KWL1	Shootin-1	BSL78_10710	2.2897663	0.002666011
A0A2G8L6W7	Oxysterol-binding protein (Fragment)	BSL78_07079	2.186826	0.001364082
**A0A2G8LLT5**	**Putative non-lysosomal glucosylceramidase-like**	**BSL78_01955**	**2.1860084**	**0.00041568**
A0A2G8JX32	Sod_Cu domain-containing protein	BSL78_22860	1.9815716	0.002369186
A0A2G8LGR0	Fibronectin type-III domain-containing protein	BSL78_03649	1.9678812	0.005892457
**A0A076VCY3**	**Glutamine synthetase (Fragment)**	**–**	**1.9303008**	**0.000025527**
A0A2G8LLG3	Triple helix repeat-containing collagen	BSL78_01933	1.7659548	0.004841135
A0A2G8JNQ4	U1 small nuclear ribonucleoprotein C (Fragment)	BSL78_25823	1.7304518	0.023951747
**A0A2G8KID3**	**Putative RWD domain-containing protein 1-like**	**BSL78_15390**	**1.6945312**	**0.000449897**
A0A2G8LG45	Collagen IV NC1 domain-containing protein	BSL78_03862	1.6842936	0.022317433
A0A2G8KTF5	Putative basement membrane-specific heparan sulfate proteoglycan core protein-like	BSL78_11836	1.663195	0.010480141
A0A2G8KPE6	Apple domain-containing protein	BSL78_13255	1.6576526	0.042417319
A0A2G8JE89	Putative cytochrome P450 1B1	BSL78_29145	1.6559028	0.035737598
A0A2G8KYU4	AIG1-type G domain-containing protein	BSL78_09939	1.6480103	0.00349669
A0A2G8JNH6	Complement component 3–2	BSL78_25841	1.637664	0.003597886
A0A2G8JMP1	B box-type domain-containing protein	BSL78_26158	1.6322986	0.032977225
A0A2G8JKB7	Putative cholesterol 24-hydroxylase isoform X2 (Fragment)	BSL78_26988	1.6132179	0.001378051
A0A2G8K5J9	Putative cAMP-dependent protein kinase type I-beta regulatory subunit isoform X2	BSL78_19858	1.606407	0.002980552
**A0A2G8KKG4**	**Putative heme-binding protein 2**	**BSL78_14698**	**1.6013692**	**0.000063966**
**A0A2G8JKA9**	**Proteoglycan 4-like**	**BSL78_27005**	**1.5897043**	**0.000097181**
A0A2G8LAW8	Putative sulfide:quinone oxidoreductase, mitochondrial	BSL78_05719	1.586919	0.005096587
**A0A2G8KY01**	**FZ domain-containing protein**	**BSL78_10244**	**1.5847688**	**0.000020500**
**A0A2G8KRL7**	**Glutamine synthetase**	**BSL78_12499**	**1.5769088**	**0.000233989**
A0A2G8KMR8	Putative charged multivesicular body protein 5	BSL78_13821	1.5691268	0.001991138
A0A2G8L3B3	Putative endoplasmic reticulum lectin 1 isoform X1	BSL78_08340	1.56023	0.022283438
**A0A2G8JHT5**	**VWFC domain-containing protein**	**BSL78_27887**	**1.549143**	**0.000082411**
A0A2G8JHS8	Metalloendopeptidase	BSL78_27906	1.5364148	0.007121039
**A0A1S5RQP9**	**Cathepsin B**	**–**	**1.5140098**	**0.000013730**
**Downregulated**				
A0A2G8JZ30	Putative nucleolar and coiled-body phosphoprotein 1 isoform X2	BSL78_22109	0.6558584	0.004082789
A0A2G8L9T1	Putative CLIP-associating protein 1-A	BSL78_06226	0.6461579	0.033128656
A0A2G8L8I9	Putative high-affinity choline transporter 1	BSL78_06498	0.6362511	0.001529379
A0A2G8JIF5	Putative multidrug resistance-associated protein 5 (Fragment)	BSL78_27659	0.6348853	0.017101150
**A0A141R8A8**	**Arginase**	**–**	**0.6298412**	**0.000000854**
A0A2G8K0J7	Fibrinogen-like protein A	BSL78_21629	0.6210056	0.023107870
**A0A2G8LBB9**	**Lipase**	**BSL78_05613**	**0.6168058**	**0.000165514**
**A0A0S1NFA9**	**Matrix metalloproteinase-1**	**–**	**0.609515**	**0.000003189**
A0A2G8KVL0	Putative NLR family CARD domain-containing protein 4	BSL78_11114	0.6068647	0.013997495
A0A286QZI9	NLRP3	NLRP3	0.604898	0.013718113
A0A2G8JWL8	Fibrinogen C-terminal domain-containing protein	BSL78_22970	0.603426	0.008390249
A0A2G8L9J9	Putative homeobox protein cut-like 1 isoform X5	BSL78_06144	0.5921187	0.033484337
A0A2G8L1K4	THO complex subunit 4	BSL78_09059	0.5914623	0.012541294
A0A2G8LMJ9	Putative neuroblast differentiation-associate d protein AHNAK	BSL78_01612	0.5873922	0.021246238
A0A2G8K2E0	Fibrinogen C-terminal domain-containing protein	BSL78_20968	0.5833804	0.001510117
A0A2G8KWA6	Putative serine/arginine repetitive matrix protein 1-like isoform X4 (Fragment)	BSL78_10855	0.5784819	0.006742302
A0A2G8KKI3	Putative CAD protein	BSL78_14662	0.5669795	0.008854254
A0A2G8L9T8	Cell surface protein	BSL78_06070	0.5658611	0.007775306
A0A2G8KCJ1	Putative popeye domain-containing protein 3	BSL78_17417	0.5567751	0.009209733
A0A2G8LRD2	Putative host cell factor 1 isoform X2	BSL78_00274	0.5514066	0.035516133
A0A2G8JUI9	SUEL-type lectin domain-containing protein	BSL78_23734	0.5488646	0.041804923
A0A2G8L646	Dynactin subunit 1	BSL78_07347	0.5307878	0.045093265
A0A2G8KE56	Cathepsin L-associated protein	BSL78_16878	0.5226544	0.007304419
A0A2G8JDM4	Nicotinate phosphoribosyltransferase (Fragment)	BSL78_29332	0.4626685	0.019824944
A0A2G8LAD2	Putative COP9 signalosome complex subunit 5	BSL78_05864	0.4543066	0.001192367
A0A2G8KAD6	Putative serine-threonine kinase receptor-associated protein-like	BSL78_18192	0.4311592	0.021254849
A0A2G8JSY4	Putative fatty acid-binding protein type 3-like	BSL78_24333	0.3648143	0.026813134

*Proteins displayed in **bold** type represent high-confidence DEPs that remain statistically significant after Benjamini-Hochberg false discovery rate (FDR) correction (q < 0.05).

**Fig. 3:**
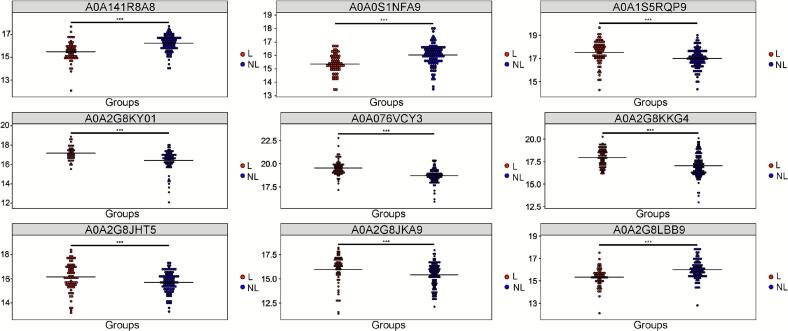
Box plots of nine statistically significant differentially expressed proteins (DEPs) (*P* < 0.01) in Liaoning versus non-Liaoning sea cucumbers (*Apostichopus japonicus*).

In addition, we further used box plots to more intuitively display the nine DEPs that were extremely significant (*P* < 0.01) between the two groups (Liaoning/non-Liaoning samples) (Fig. 3). The specific functions of some key DEPs provide direct mechanistic hypotheses for the geographical differentiation between Liaoning and non-Liaoning sea cucumbers, particularly in terms of nitrogen homeostasis, structural integrity, and lipid metabolism.

**Nitrogen metabolism and stress adaptation.**

The marked downregulation of arginase (A0A141R8A8) alongside the upregulation of glutamine synthetase (A0A076VCY3 and BSL78_12499) delineates a concerted reprogramming of nitrogen metabolism in Liaoning samples. The pronounced decrease in arginase (fold change = 0.63), a pivotal enzyme diverting arginine towards ornithine and urea, may serve a dual adaptive purpose. Firstly, by reducing substrate flux into nitric oxide (NO) synthesis, it could alleviate potential nitrosative stress under pronounced environmental fluctuations (Lee & Chen, 2004). Secondly, its downregulation aligns with reports that arginase activity increases with pathogen challenge (Yina et al., 2016); thus, lower expression may correlate with a reduced pathogen burden in Liaoning specimens, indicating a healthier or more resilient state. Concurrently, enhanced glutamine synthesis bolsters cellular osmoprotection and ammonia detoxification (Wang et al., 2014). This coordinated shift, suppressing one branch while augmenting another, likely establishes a more robust and efficient nitrogen homeostasis, forming a core metabolic adaptation underpinning the stress resilience observed in Liaoning sea cucumbers. Separately, the significant upregulation of the Frizzled domain-containing protein A0A2G8KY01 (BSL78_10244, fold change = 1.58) hints at differentiated developmental or tissue-regenerative processes, as Frizzled proteins are implicated in intestinal regeneration in sea cucumbers.

**Collagen stability and texture.**

The significant downregulation of matrix metalloproteinase-1 (MMP-1/Collagenase, A0A0S1NFA9) provides a direct molecular rationale for the superior texture prized in Liaoning sea cucumbers. As a key enzyme responsible for initiating the degradation of collagen, the suppressed expression of MMP-1 implies a reduced turnover rate of the collagenous network within the body wall (Chen et al., 2022). This decline in collagenolytic activity promotes the accumulation and enhances the structural stability of collagen, the primary component determining texture. Consequently, this finding offers a precise proteomic explanation for the consistently reported higher collagen integrity and firmer texture of Liaoning-origin *A. japonicus* (Zhang et al., 2023).

**Lipid metabolism and nutritional flavor.**

The differential expression of lipase (A0A2G8LBB9), which maps to lipid metabolism pathways, suggests potential modifications in lipid utilization or signaling mechanisms. Altered lipase activity could significantly influence the profile of free fatty acids, molecules that serve dual roles as vital energy substrates and as precursors for flavor compounds. This reprogramming of lipid metabolism may therefore partially explain the distinct nutritional profiles and flavor perception reported for sea cucumbers from different geographical regions (Feng et al., 2021), warranting further targeted metabolomic investigation to elucidate the specific lipid-derived compounds involved.

Unfortunately, other DEPs like A0A2G8KKG4 (BSL78_14698), A0A2G8JHT5 (BSL78_27887) and A0A2G8JKA9 (BSL78_27005) could not be fully characterized so far in sea cucumbers due to the lack of relevant studies. To generate further hypotheses, we performed additional analysis which confirmed the SOUL heme-binding protein domain in A0A2G8KKG4, supporting a role in heme binding (Table 1). For A0A2G8JHT5 and A0A2G8JKA9, our analysis did not yield significant homology results or informative domain annotations.

While these individual DEPs provide mechanistic insights into the geographical proteomic differences between Liaoning and non-Liaoning samples, the point-like analysis could not reflect the entire biological role, therefore we will subsequently employ Gene Ontology annotation (GO) and Kyoto Encyclopedia of Genes and Genomes (KEGG) pathway mapping to systematically elucidate the potential bioinformatics functions of these DEPs.

### 3.3 Gene ontology (GO) annotation analysis of DEPs

**Fig. 4:**
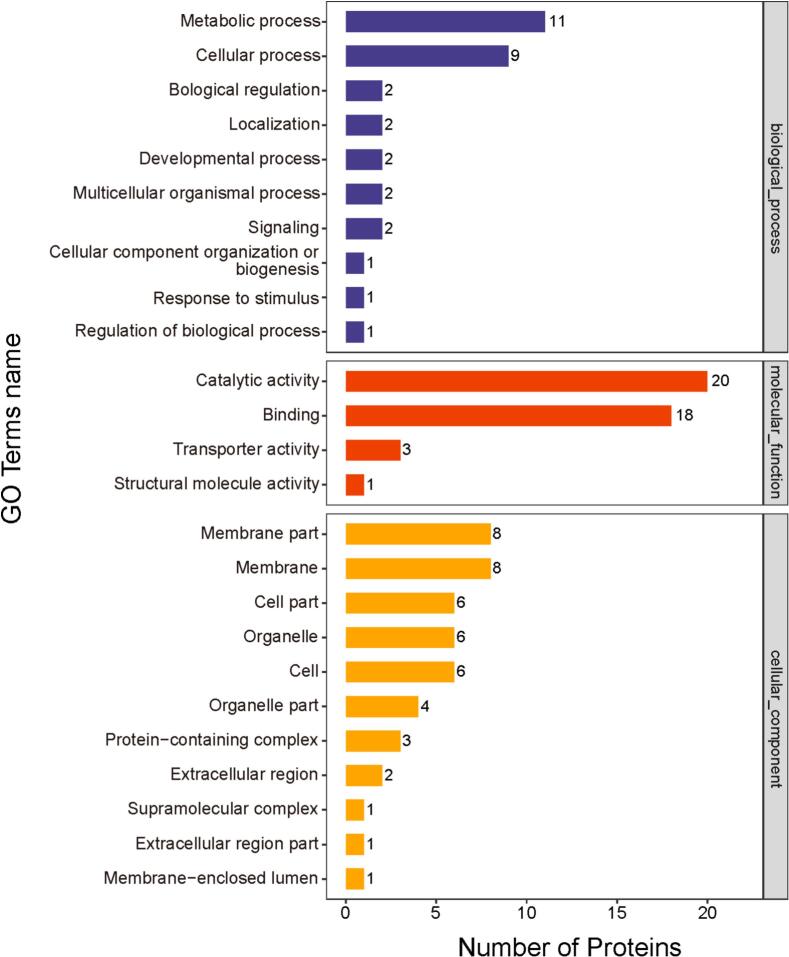
Gene Ontology (GO) annotation statistics of differentially expressed proteins (DEPs) in Liaoning versus non-Liaoning sea cucumbers (*Apostichopus japonicus*) across biological process (BP), molecular function (MF) and cellular component (CC) categories.

The Gene Ontology (GO) annotation of DEPs between Liaoning and non-Liaoning samples revealed distinct functional hierarchies across biological process, molecular function, and cellular component. In biological processes, metabolic processes (11 DEPs) and cellular processes (9 DEPs) constituted the predominant categories, collectively accounting for 61% of annotated proteins (Fig. 4). This prominence suggests geographical variations may significantly influence fundamental cellular maintenance and energy metabolism pathways, potentially reflecting adaptive responses to divergent environmental conditions or nutrient availability between regions. Similarly, previous studies have shown that cellular metabolic processes can be significantly impacted by aquatic environments such as water temperature (Doney et al., 2012; Hoegh-Guldberg & Bruno, 2010; Priya et al., 2023), so the fact that sea cucumbers in Liaoning waters grow in waters with large temperature differences may explain our findings. In agreement with that, the GO annotation results of sea cucumber by Feng et al. (2020) also showed that “metabolic process” and “cellular process” dominated the proteins identified in the biological process (> 50%), and Zeng et al. (2024) found through clustering hierarchical analysis and GO term enrichment analysis that proteins related to the expression of “metabolic process” dominated the intestinal regeneration of sea cucumbers, which indicated that metabolic proteins played an important role in the development of organisms.

At the molecular function level, catalytic activity (20 DEPs) and binding capacity (18 DEPs) emerged as dominant features, comprising 91% of all annotated functions (Fig. 4). This enzymatic predominance aligns with the observed metabolic process enrichment, suggesting coordinated regulation of biochemical networks. The presence of transporter proteins (3 DEPs) further supports potential adaptations in membrane-mediated substance exchange, possibly linked to osmoregulatory demands in distinct marine habitats (Jiang et al., 2024).

Cellular component analysis demonstrated membrane system specialization, with membrane parts (8 DEPs) and membrane-associated proteins (8 DEPs) constituting 35% of compartmentalized DEPs (Fig. 4). This membrane-centric distribution implies geographical differences may particularly affect cellular signaling and transmembrane transport mechanisms, which echoed the findings in the “Biological Processes” and “Molecular Functions” discussed above. Furthermore, the detection of extracellular matrix components (3 DEP) may suggest potential differences in the collagen network structure of the body wall of sea cucumbers from the two origins.

Furthermore, GO enrichment analysis using Fisher's Exact Test confirmed that “glutamine family amino acid metabolic processes” was the most significantly enriched among the biological process categories (**Fig. S4**), which was consistent with the previously observed in **section 3.2** dominance of upregulated stress-responsive family proteins A0A076VCY3 and BSL78_12499 (Wang et al., 2014), suggesting the metabolic processes of glutamine family amino acid plays an important role in adaptation to regional environmental stressors. At the molecular function category, the activities of certain ligases were prominently enriched, including carbon‑nitrogen bond ligase, glutamate-ammonia ligase, ammonia ligase and acid-ammonia (or amide) ligase, which suggests the specialization of enzymes in substrate utilization in sea cucumbers of different habitats (**Fig. S5**). In terms of cellular components, kinetochore microtubule, spindle microtubule, and cytoplasmic microtubule are more prominent, but their number is small, so they do not provide more valuable information (**Fig. S6**).

### 3.4 KEGG pathway analysis of DEPs

**Fig. 5:**
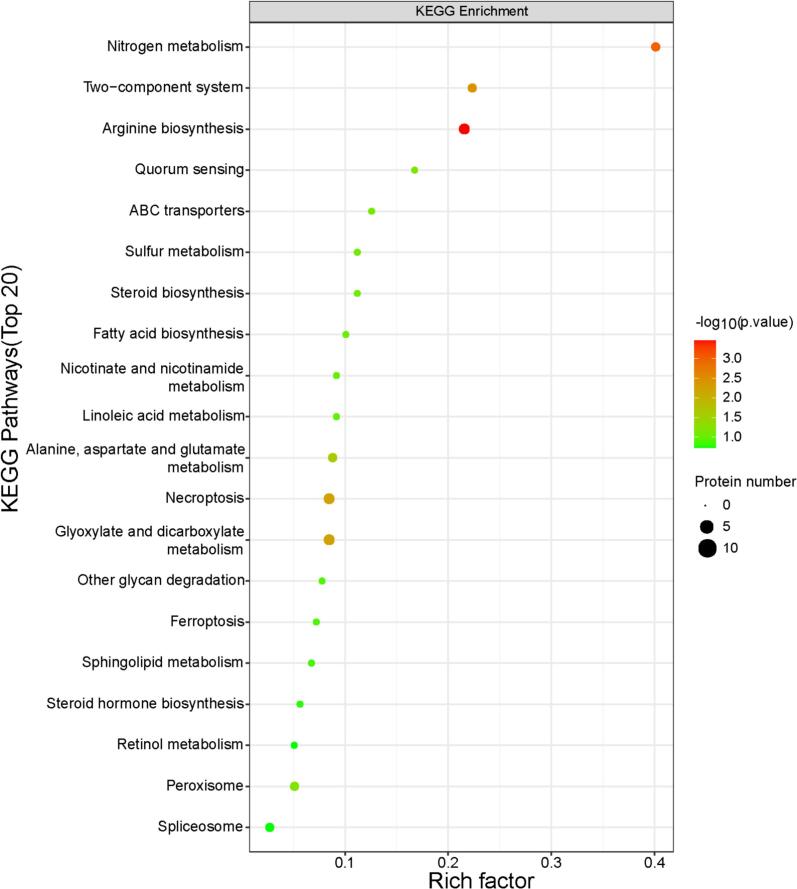
Bubble chart of enrichment of differentially expressed proteins (DEPs) in Kyoto Encyclopedia of Genes and Genomes (KEGG) pathways of Liaoning versus non-Liaoning sea cucumbers (*Apostichopus japonicus*). Bubble size reflects DEP count; colour indicates enrichment significance; Rich factor = (DEPs in pathway) / (Total proteins in pathway).

KEGG pathway analysis of differentially expressed proteins (DEPs) revealed significant enrichment (*P* < 0.05) in three primary categories: metabolism, environmental information processing and cellular processes (Level 1 classification). Further Level 2 classification identified key sub-pathways, namely i) Metabolism: arginine biosynthesis (3 DEPs, *P* = 0.00034), nitrogen metabolism (2 DEPs, *P* = 0.0010), alanine/aspartate/glutamate metabolism (2 DEPs, *P* = 0.023) and glyoxylate/dicarboxylate metabolism (3 DEPs, *P* = 0.0057); ii) Environmental information processing: two-component system (2 DEPs, *P* = 0.0035); iii) Cellular processes: necroptosis (3 DEPs, *P* = 0.0057) (Fig. 5).

The marked enrichment of arginine biosynthesis suggests its potential pivotal role in biogeographical adaptation. This pathway integrates nitrogen homeostasis via urea cycle intermediates, redox balance through nitric oxide (NO) signaling, and energy buffering via phosphocreatine synthesis, these functions are critical for marine organisms facing environmental heterogeneity (Fung et al., 2025; Yina et al., 2016), suggesting Liaoning specimens may optimize arginine metabolism to counterbalance colder water-induced metabolic constraints. Furthermore, the subsidiary pathway enrichments reveal systemic adaptation strategies. Firstly, the co-enrichment of nitrogen metabolism and alanine/aspartate/glutamate metabolism complements arginine biosynthesis by facilitating ammonia detoxification and carbon skeleton recycling. Secondly, the significant differences in glyoxylate/dicarboxylate metabolism supported carbohydrate metabolic reprogramming and TCA cycle replenishment, mirroring hypoxia adaptation mechanisms in marine invertebrates such as hard clam (*Mercenaria mercenaria*) and sea cucumber (*Apostichopus japonicus*) (Hu et al., 2022; Li et al., 2019). Therefore, this activation of this pathway may enhance oxygen-independent ATP production, potentially compensating for the lower seawater temperature in Liaoning.

Beyond arginine-centric metabolic reprogramming, the significant enrichment of two-component systems in Liaoning samples reflects enhanced environmental sensing capabilities, likely driven by the extreme annual temperature range (ΔT ≈ 25 °C) in Bohai Sea (Hu et al., 2022). This adaptation may preserve metabolic homeostasis, which could contribute to maintaining texture-related proteins (e.g., collagen) under thermal stress, as inferred from the documented texture superiority of Liaoning specimens (Clark & Peck, 2009). It is important to note that the database search was performed against a host (*A. japonicus*) specific proteome. The enrichment of two-component system pathway was originally described in bacteria, but this reflects the potential presence of evolutionarily conserved signaling components in eukaryotes. Additionally, significant differences in necroptosis, a regulated inflammatory cell death pathway, may reflect tissue remodeling or immune responses to region-specific pathogens, as Liaoning sea cucumbers reportedly inhabit colder waters with different sediment microbiomes and may therefore employ necroptosis to locally suppress microbial threats without triggering systemic inflammation (Virolle et al., 2019).

The pathway enrichment analysis provides a systemic context that substantiates the functional hypotheses derived from key individual DEPs. The significant enrichment of the arginine biosynthesis and nitrogen metabolism pathways offers strong corroboration for the observed coordinated downregulation of arginase and upregulation of glutamine synthetase, framing these changes as part of a broader metabolic reprogramming essential for nitrogen homeostasis and stress adaptation. Similarly, the enrichment of the two-component system pathway aligns with the proposed enhancement of environmental sensing capabilities in Liaoning samples. While not a top-level KEGG pathway, the critical downregulation of MMP-1 (A0A0S1NFA9) can be understood as a decisive modulation within the extracellular matrix (ECM) degradation network, directly impacting the collagen stability pathway. The involvement of lipid metabolism, suggested by the DEP lipase, is reflected in the enrichment of related metabolic categories. A comprehensive overview of up- and down-regulated proteins across all enriched pathways is provided in **Fig. S7**. This confluence of evidence from both discrete protein markers and integrated pathway shifts indicates that the geographical differentiation of *A. japonicus* is orchestrated through specific regulatory proteins embedded within larger adaptive metabolic and signaling networks.

It is noteworthy that the “non-Liaoning” group comprised samples from both Shandong and Fujian provinces. Although this grouping is pragmatic for the primary authentication question (Liaoning vs. others), it may introduce additional heterogeneity, as these regions possess distinct oceanographic conditions. Therefore, while the DEPs identified herein are robust for distinguishing Liaoning specimens, the markers for precisely discriminating Shandong from Fujian origins warrant further investigation with a dedicated study design and larger sample sizes per province. In addition, this study was designed to investigate geographic origin under practical, commercial conditions. As such, variables such as detailed feeding regimes and feed composition at individual farms were not characterized. While these factors could contribute to some within-group variance, our large-scale sampling from multiple farms within each region and the clear separation by geographic origin in our PCA analysis suggest that the signal of geographic origin is robust and dominant. Future studies employing controlled feeding trials would be valuable to further dissect the specific contributions of diet versus terroir.

## 4 Conclusions

In this study, 200 sea cucumber samples from Liaoning, Shandong, and Fujian provinces were analyzed using a customized label-free DDA library-based DIA workflow to characterize the proteomic profiles underlying the geographic differentiation of Liaoning sea cucumbers from those harvested in Shandong and Fujian, China. The identification of 64 DEPs and pathway-level enrichment of arginine biosynthesis highlighted the unique nitrogen metabolism of Liaoning samples, which may be key to their adaptation to colder, nutrient-fluctuating habitats. Significant differential enrichment of two-component systems and necroptosis pathways further highlighted possible unique adaptive strategies for Liaoning sea cucumber: i) core metabolic homeostasis via arginine-mediated urea/NO signaling, ii) dynamic environmental sensing via bacteria-derived phosphorylation cascades, and iii) local pathogen control via controlled cell death. These findings generate testable mechanistic hypotheses to explain the market distinction of Liaoning sea cucumbers, proposing links between proteomic features and key traits such as texture (collagen stability) and nutritional quality (lipid metabolism). The DIA-based workflow established here enables high-throughput discovery and screening of potential geographical biomarker candidates. This provides a solid data foundation and methodological basis for future research aimed at developing validated tools to combat sea cucumber fraud such as the mislabeling of Liaoning products with those from Shandong or Fujian.

It is important to note that the proposed links between proteomic signatures and commercial quality traits (e.g., texture) are based on associations inferred by integrating our data with established literature. This study provides a set of high-confidence molecular candidates and mechanistic hypotheses. Future work that directly integrates proteomic profiling with quantitative measurements of texture, collagen properties, and environmental parameters in the same sample cohort is essential to validate these proposed mechanisms and establish causal relationships.

## CRediT authorship contribution statement

**Qiong Wu:** Writing – review & editing, Writing – original draft, Visualization, Investigation, Funding acquisition, Formal analysis, Conceptualization. **Xiliang Yu:** Writing – review & editing, Methodology. **Shiwei Wan:** Writing – review & editing, Methodology. **Houde Cai:** Writing – review & editing, Methodology. **Jian Jiao:** Writing – review & editing, Resources, Conceptualization. **Jiaojiao Shi:** Writing – review & editing, Methodology, Formal analysis. **Xiuping Dong:** Writing – review & editing, Visualization, Supervision, Resources, Funding acquisition, Conceptualization.

## Declaration of competing interest

The authors declare that they have no known competing financial interests or personal relationships that could have appeared to influence the work reported in this paper.

## Data Availability

The mass spectrometry proteomics data generated in this study have been deposited to the ProteomeXchange Consortium via the iProX partner repository with the dataset identifier PXD072020.

## Appendix A Supplementary data

Supplementary material Image 1
