# Supplementary material for: Label-free quantitative proteomics analysis of sea cucumber (Apostichopus japonicus) from different origins using data-independent acquisition mass spectrometry
Source: Food Chem X. 2026 Feb 13;34:103666. doi: 10.1016/j.fochx.2026.103666 (PMC12924188; doi:10.1016/j.fochx.2026.103666)

**Supplementary materials of:**

**Label-free quantitative proteomics analysis of sea cucumber (*Apostichopus japonicus*) from different origins using data-independent acquisition mass spectrometry**

Qiong Wu^a^, Xiliang Yu ^a^, Shiwei Wan^a^, Houde Cai^a^, Jian Jiao^b^, Jiaojiao Shi^c^, Xiuping Dong^a^*

^a^ State Key Lab of Marine Food Processing & Safety Control, National Engineering Research Center of Seafood, School of Food Science and Technology, Dalian Polytechnic University, 116034 Dalian, P. R. China.

^b^ Beijing Tong Ren Tang Health (Dalian) Seafoods Co. L td., 116045 Dalian, P. R. China.

^c^ Shanghai Applied Protein Technology Co. L td., 201100 Shanghai, P. R. China.

*Corresponding author

E-mail: [dxiuping@163.com](mailto:dxiuping@163.com)

Tel:+86-411-86318785

Fax:+86-411-86323671

**Table S1.** Sample information in this study.

| **Group** | ***n*** | **Sampling date** | **Average weight (g)** | **Location** | **Latitude (N)** | | **Longitude (E)** |
| --- | --- | --- | --- | --- | --- | --- | --- |
| Liaoning | 20 | May 2022 | 113.7 ± 26.7 | Liaoning | 39.2931 | 121.6842 | |
|  | 20 | May 2022 |  | Liaoning | 39.4550 | 122.5082 | |
|  | 20 | May 2022 |  | Liaoning | 39.2965 | 121.5098 | |
|  | 20 | May 2022 |  | Liaoning | 39.7359 | 123.4111 | |
| Non-liaoning | 20 | March 2022 | 82.2 ± 25.0 | Fujian | 26.3195 | 119.8483 | |
|  | 20 | March 2022 |  | Fujian | 26.2842 | 119.7808 | |
|  | 20 | March 2022 |  | Fujian | 26.6547 | 119.8951 | |
|  | 20 | May 2022 |  | Shandong | 36.4965 | 120.7960 | |
|  | 20 | May 2022 |  | Shandong | 36.6778 | 121.2375 | |
|  | 20 | May 2022 |  | Shandong | 37.9087 | 120.7275 | |

**Table S2.** Chemicals used in this study.

| **Chemical** | **Cat. No** | **Manufacturer** |
| --- | --- | --- |
| Urea | 161‐0731 | Bio‐Rad |
| Tris | A6141 | Sigma |
| HCl | 10011018 | Sinopharm |
| BCA Protein Assay Kit | P0012 | Beyotime |
| SDS | 161‐0302 | Bio‐Rad |
| Dithiothreitol (DTT) | 161‐0404 | Bio‐Rad |
| Iodoacetamide (IAA) | 163‐2109 | Bio‐Rad |
| Trypsin | 317107 | Promega |
| C18 Cartridge | 66872‐U | Sigma |
| Formic Acid (FA) | 06450 | Fluka |

**Table S3.** BH-adjusted *P* values for the originally identified 64 DEPs.

| **Protein ID** | **Gene Name** | **BH-adjusted *P* value** |
| --- | --- | --- |
| H9TUY5 | RPL32e | 0.185504427 |
| A0A2G8LA92 | BSL78_05931 | 0.263568473 |
| A0A2G8L691 | BSL78_07341 | 0.355763622 |
| A0A2G8JYL3 | BSL78_22296 | 0.348030399 |
| A0A2G8L0Q1 | BSL78_09315 | 0.2911152 |
| A0A2G8LDD8 | BSL78_04824 | 0.320778931 |
| A0A2G8L3G5 | BSL78_08319 | 0.324650901 |
| A0A2G8JBG1 | BSL78_30106 | 0.09385876 |
| A0A2G8JDJ0 | BSL78_29372 | 0.330639507 |
| A0A2G8KWL1 | BSL78_10710 | 0.106478863 |
| A0A2G8L6W7 | BSL78_07079 | 0.080322422 |
| A0A2G8LLT5 | BSL78_01955 | 0.039922905 |
| A0A2G8JX32 | BSL78_22860 | 0.103610349 |
| A0A2G8LGR0 | BSL78_03649 | 0.159580639 |
| A0A076VCY3 | - | 0.010913102 |
| A0A2G8LLG3 | BSL78_01933 | 0.143296848 |
| A0A2G8JNQ4 | BSL78_25823 | 0.280192918 |
| A0A2G8KID3 | BSL78_15390 | 0.041013393 |
| A0A2G8LG45 | BSL78_03862 | 0.267793518 |
| A0A2G8KTF5 | BSL78_11836 | 0.207192381 |
| A0A2G8KPE6 | BSL78_13255 | 0.355763622 |
| A0A2G8JE89 | BSL78_29145 | 0.331705308 |
| A0A2G8KYU4 | BSL78_09939 | 0.128017689 |
| A0A2G8JNH6 | BSL78_25841 | 0.129327645 |
| A0A2G8JMP1 | BSL78_26158 | 0.324234417 |
| A0A2G8JKB7 | BSL78_26988 | 0.080322422 |
| A0A2G8K5J9 | BSL78_19858 | 0.113318285 |
| A0A2G8KKG4 | BSL78_14698 | 0.020393473 |
| A0A2G8JKA9 | BSL78_27005 | 0.022603213 |
| A0A2G8LAW8 | BSL78_05719 | 0.145796747 |
| A0A2G8KY01 | BSL78_10244 | 0.010132202 |
| A0A2G8KRL7 | BSL78_12499 | 0.029844868 |
| A0A2G8KMR8 | BSL78_13821 | 0.09385876 |
| A0A2G8L3B3 | BSL78_08340 | 0.267793518 |
| A0A2G8JHT5 | BSL78_27887 | 0.02126175 |
| A0A2G8JHS8 | BSL78_27906 | 0.172739809 |
| A0A1S5RQP9 | - | 0.010132202 |
| A0A2G8JZ30 | BSL78_22109 | 0.132322513 |
| A0A2G8L9T1 | BSL78_06226 | 0.324234417 |
| A0A2G8L8I9 | BSL78_06498 | 0.083578449 |
| A0A2G8JIF5 | BSL78_27659 | 0.245883448 |
| A0A141R8A8 | - | 0.00337585 |
| A0A2G8K0J7 | BSL78_21629 | 0.272741851 |
| A0A2G8LBB9 | BSL78_05613 | 0.028453988 |
| A0A0S1NFA9 | - | 0.006304796 |
| A0A2G8KVL0 | BSL78_11114 | 0.237536892 |
| A0A286QZI9 | NLRP3 | 0.234811342 |
| A0A2G8JWL8 | BSL78_22970 | 0.18742962 |
| A0A2G8L9J9 | BSL78_06144 | 0.324650901 |
| A0A2G8L1K4 | BSL78_09059 | 0.22957536 |
| A0A2G8LMJ9 | BSL78_01612 | 0.263568473 |
| A0A2G8K2E0 | BSL78_20968 | 0.083578449 |
| A0A2G8KWA6 | BSL78_10855 | 0.172225841 |
| A0A2G8KKI3 | BSL78_14662 | 0.196683827 |
| A0A2G8L9T8 | BSL78_06070 | 0.178764722 |
| A0A2G8KCJ1 | BSL78_17417 | 0.197909156 |
| A0A2G8LRD2 | BSL78_00274 | 0.330639507 |
| A0A2G8JUI9 | BSL78_23734 | 0.355763622 |
| A0A2G8L646 | BSL78_07347 | 0.362395873 |
| A0A2G8KE56 | BSL78_16878 | 0.173906163 |
| A0A2G8JDM4 | BSL78_29332 | 0.258521747 |
| A0A2G8LAD2 | BSL78_05864 | 0.076042273 |
| A0A2G8KAD6 | BSL78_18192 | 0.263568473 |
| A0A2G8JSY4 | BSL78_24333 | 0.298096407 |

**Table S4. Quality control metrics for the DIA quantitative analysis.**

| **Metric** | **Value / Description** |
| --- | --- |
| Total proteins identified (FDR<1%) | 6,278 |
| Missing Values | 30% |
| **QC Sample Reproducibility** |  |
| Number of QC injections | 29 |
| Median Coefficient of Variation (CV) across QC runs | ~30% |
| Assessment method | PCA clustering (**Fig. S1**) & CV analysis |

**Table S5.** Comparison of key proteomic metrics between the present DIA workflow and previous studies on sea cucumber.

| Methods | Sample Size | Number of proteins identified | Number of peptides identified | Reference |
| --- | --- | --- | --- | --- |
| Customized DIA workflow | 200 | 6,278 | 78227 | This study |
| SWATH | 72 | 548 | 13194 | Jiang et al., 2021 |
| MaxQuant | 12 | 376 | - | Gu et al., 2022 |
| TMT | 30 | 5051 | 25899 | Feng et al., 2020 |
| iTRAQ | 18 | 4073 | 20214 | Sun et al., 2017 |

**Figure S1.** Three-dimensional PCA score plot of experimental and QC samples. Color coding: green (non-Liaoning origin), blue (Liaoning origin), pink (QC).


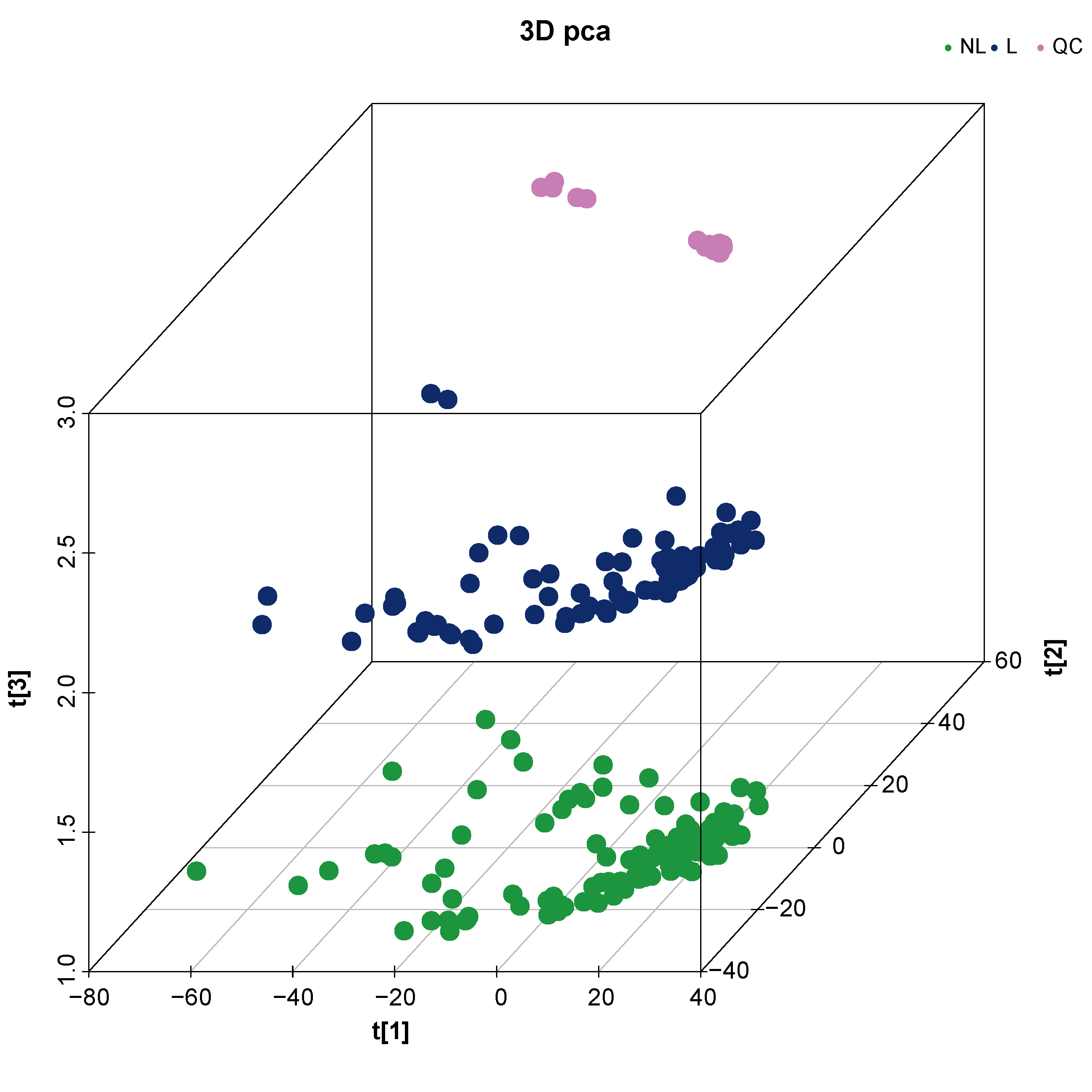


**Figure S2.** Average data point distribution of chromatographic peaks.


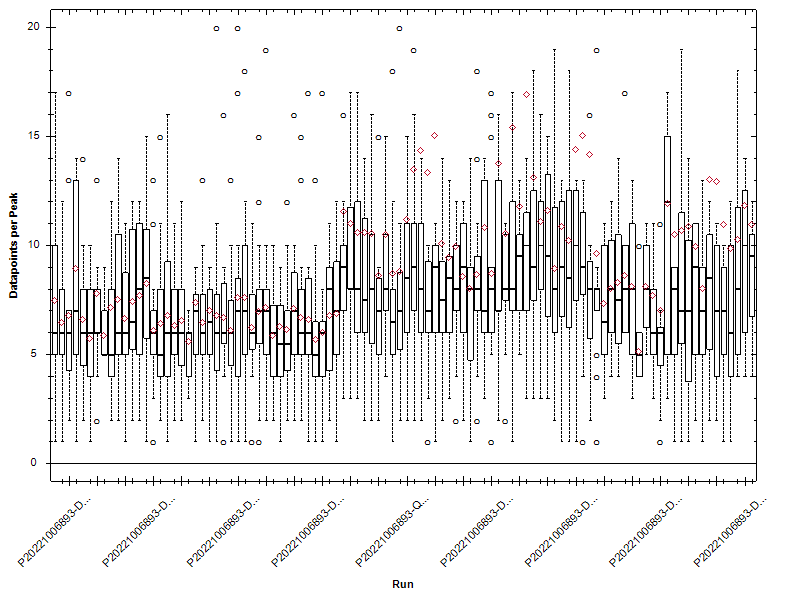


**Figure S3.** Statistical graph of column peak capacity in DIA experiment.


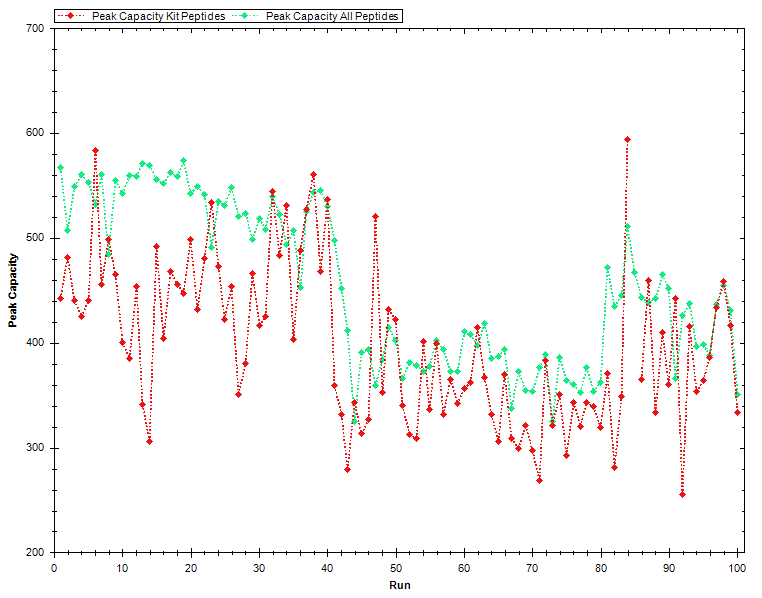


**Figure S4.** Bubble diagram of GO function enrichment in biological process classification of Liaoning versus non-Liaoning sea cucumbers (*Apostichopus japonicus*).


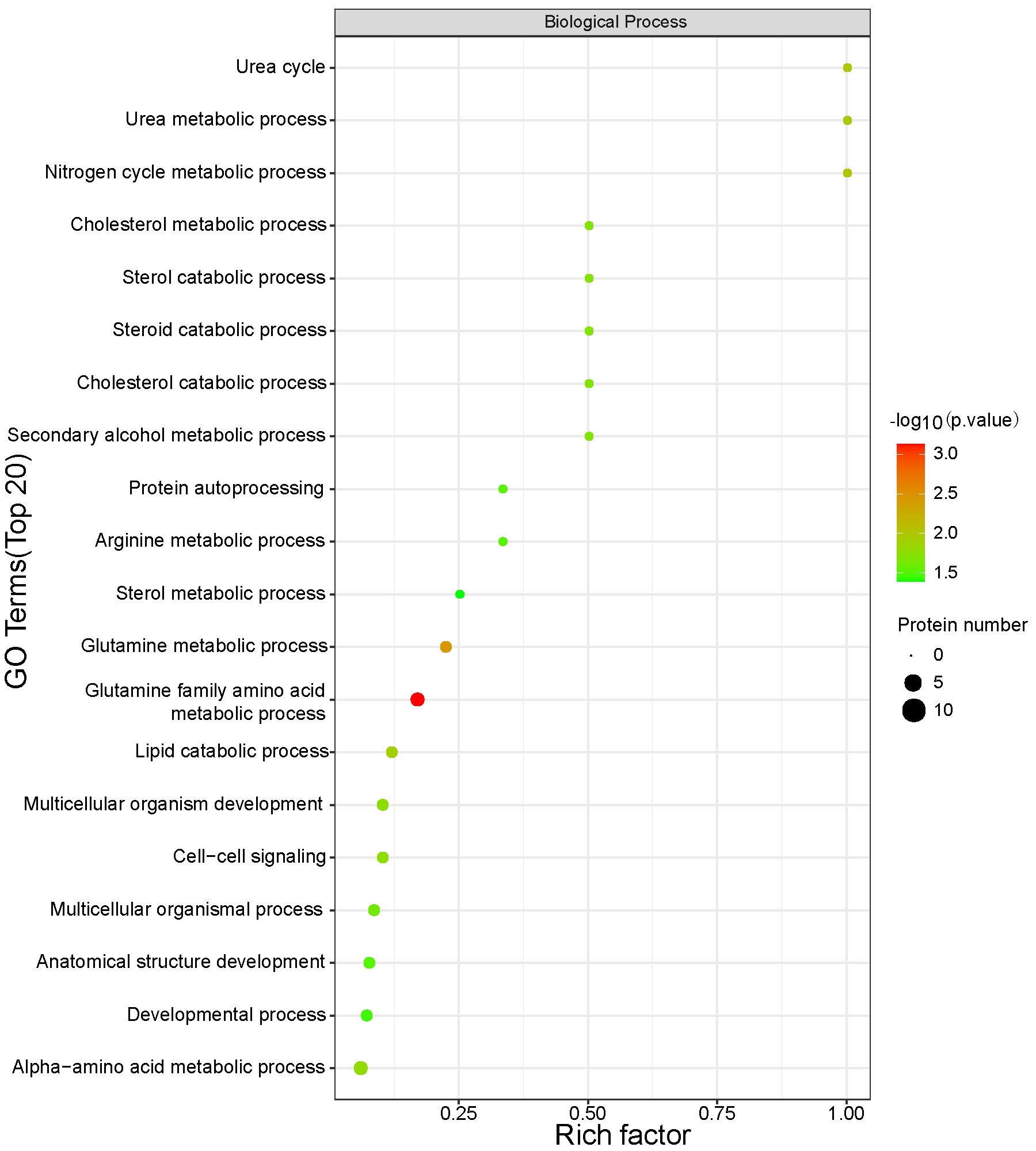


**Figure S5.** Bubble diagram of GO function enrichment in molecular function classification of Liaoning versus non-Liaoning sea cucumbers (*Apostichopus japonicus*).


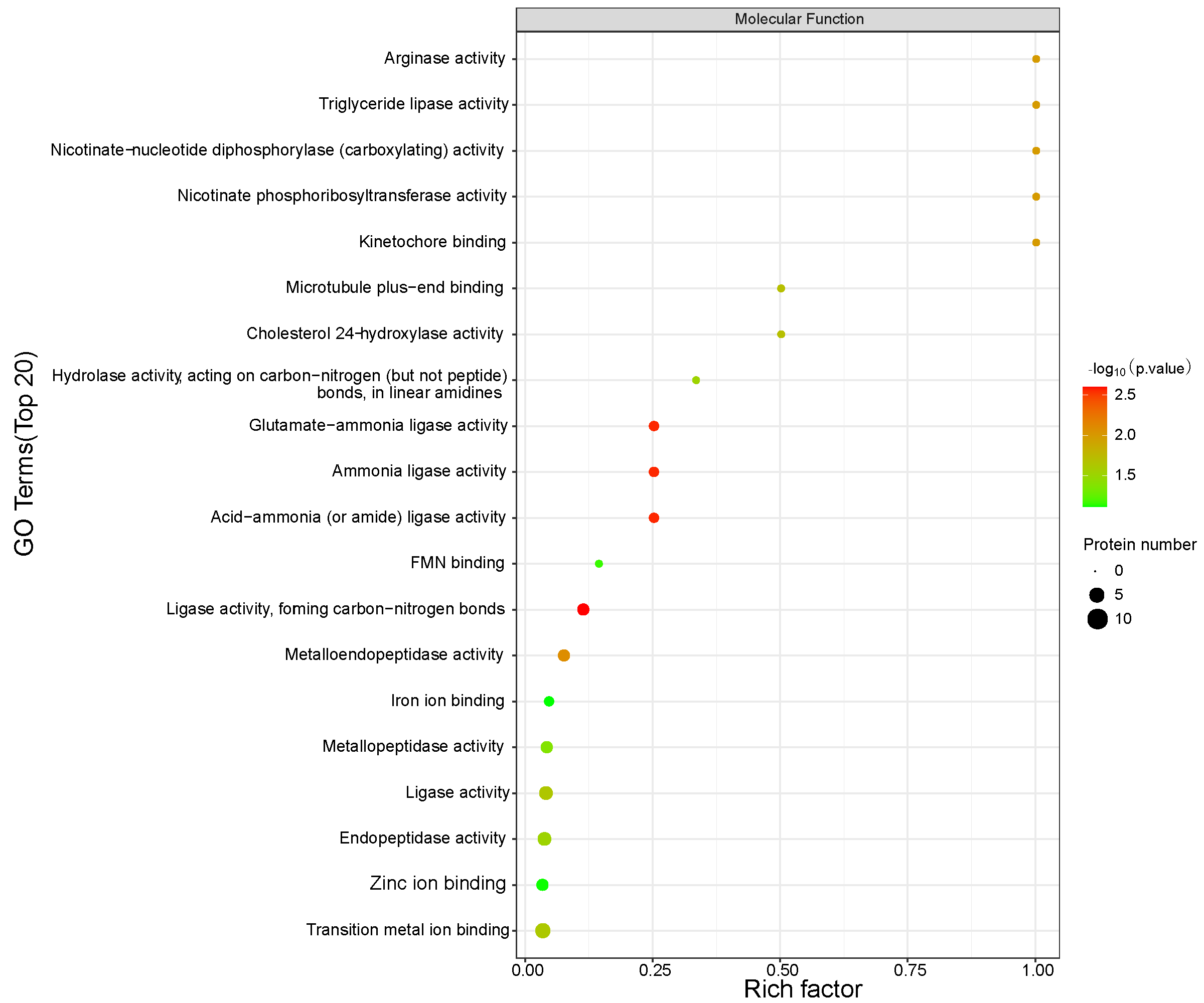


**Figure S6.** Bubble diagram of GO function enrichment in cellular component classification of Liaoning versus non-Liaoning sea cucumbers (*Apostichopus japonicus*).


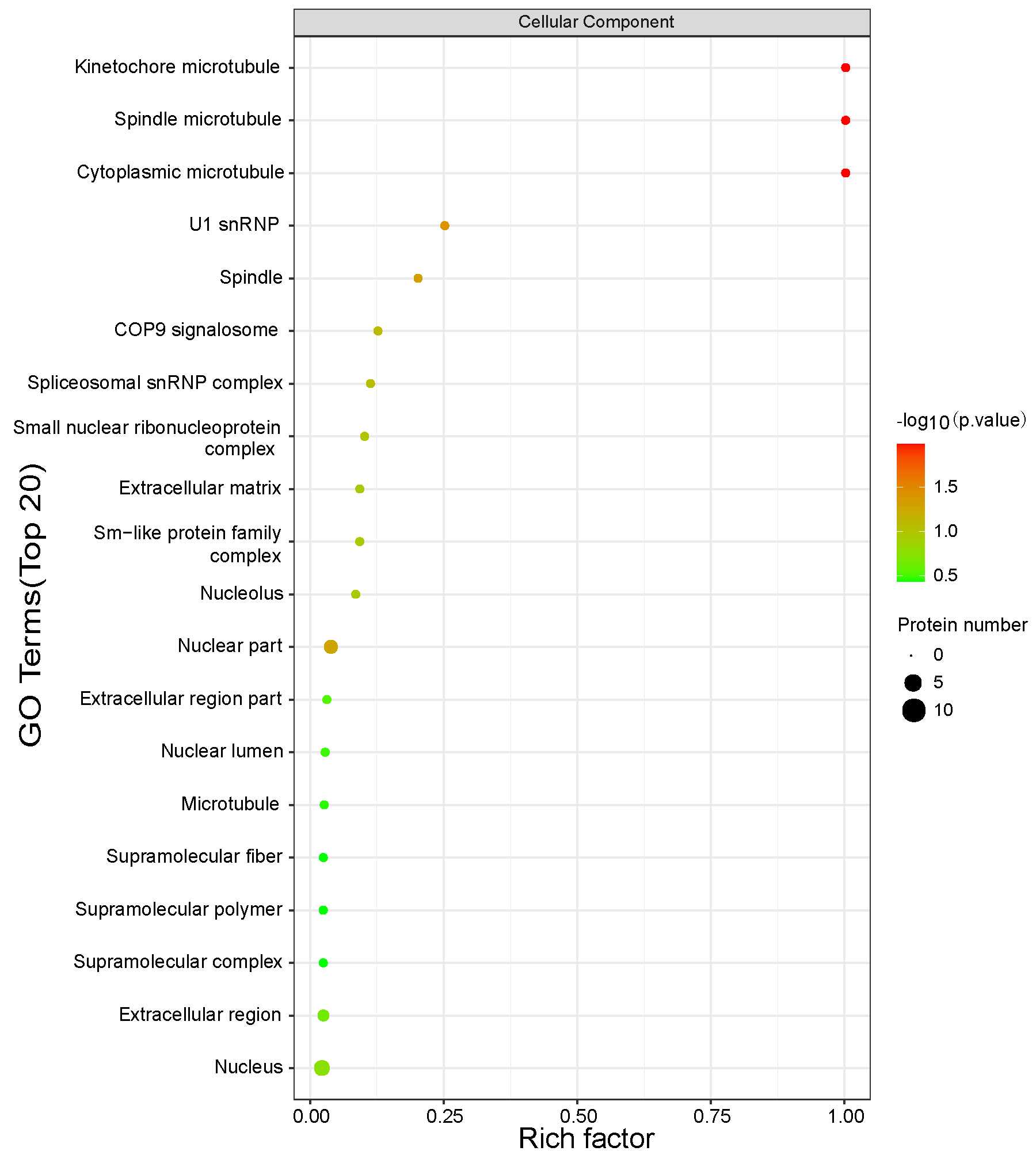


**Figure S7.** Butterfly plot of KEGG pathway enrichment for DEPs of Liaoning versus non-Liaoning sea cucumbers (*Apostichopus japonicus*).


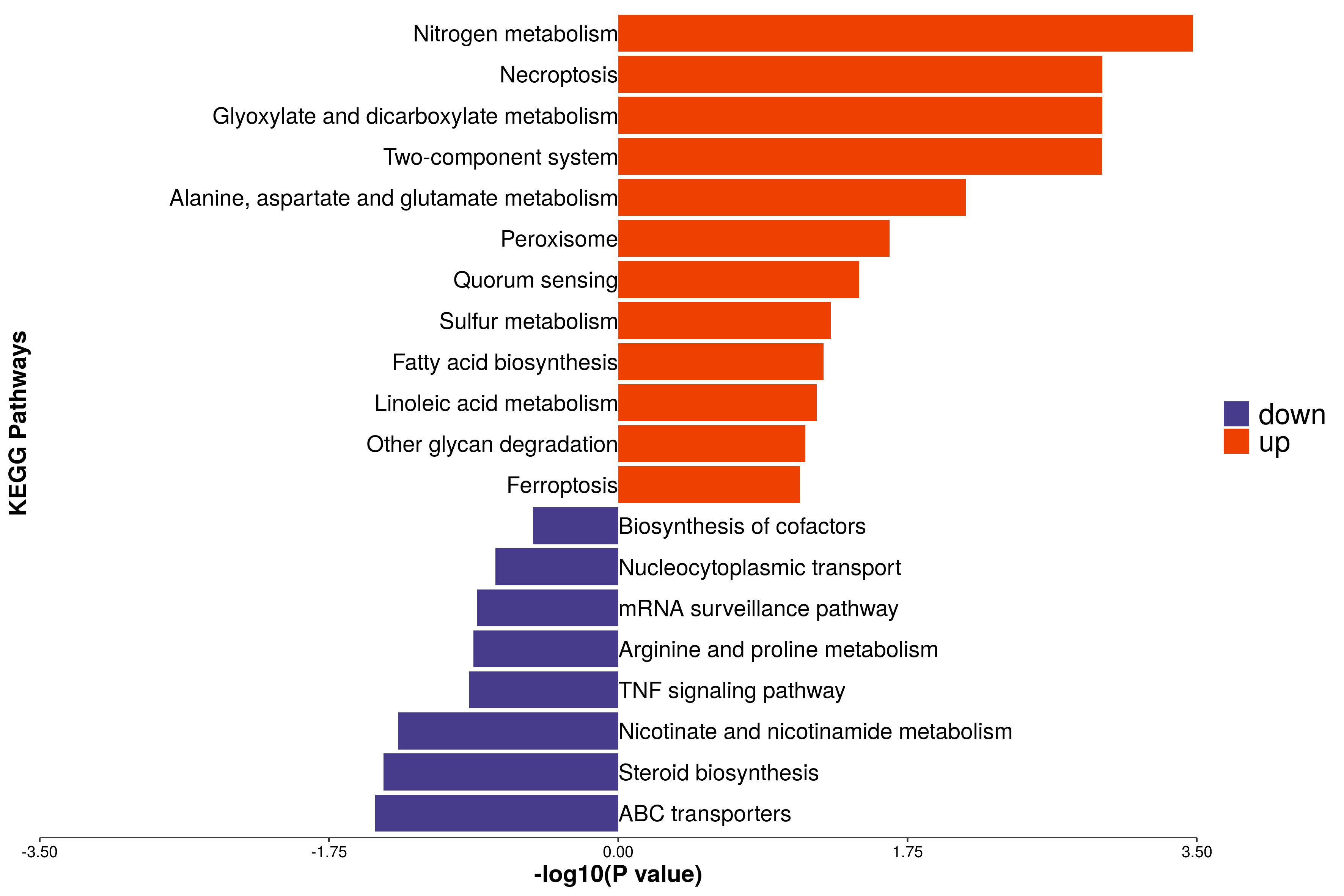

Supplement: Supplementary file 1 — Supplementary material [file mmc1.docx]
